# NKCC1-Deficiency Results in Abnormal Proliferation of Neural Progenitor Cells of the Lateral Ganglionic Eminence

**DOI:** 10.3389/fncel.2016.00200

**Published:** 2016-08-17

**Authors:** Ana Cathia Magalhães, Claudio Rivera

**Affiliations:** ^1^Neuroscience Center, University of HelsinkiHelsinki, Finland; ^2^Aix-Marseille University, UMR S901Marseille, France; ^3^INSERM U901, Institut de Neurobiologie de la Méditerranée (INMED)Marseille, France

**Keywords:** NKCC1, cell cycle decision, Olig2, interneuron progenitors, chloride homeostasis

## Abstract

The proliferative pool of neural progenitor cells is maintained by exquisitely controlled mechanisms for cell cycle regulation. The Na-K-Cl cotransporter (NKCC1) is important for regulating cell volume and the proliferation of different cell types *in vitro*. NKCC1 is expressed in ventral telencephalon of embryonic brains suggesting a potential role in neural development of this region. The ventral telencephalon is a major source for both interneuron and oligodendrocyte precursor cells. Whether NKCC1 is involved in the proliferation of these cell populations remains unknown. In order to assess this question, we monitored several markers for neural, neuronal, and proliferating cells in wild-type (WT) and NKCC1 knockout (KO) mouse brains. We found that NKCC1 was expressed in neural progenitor cells from the lateral ganglionic eminence (LGE) at E12.5. Mice lacking NKCC1 expression displayed reduced phospho-Histone H3 (PH3)-labeled mitotic cells in the ventricular zone (VZ) and reduced cell cycle reentry. Accordingly, we found a significant reduction of Sp8-labeled immature interneurons migrating from the dorsal LGE in NKCC1-deficient mice at a later developmental stage. Interestingly, at E14.5, NKCC1 regulated also the formation of Olig2-labeled oligodendrocyte precursor cells. Collectively, these findings show that NKCC1 serves *in vivo* as a modulator of the cell cycle decision in the developing ventral telencephalon at the early stage of neurogenesis. These results present a novel mechanistic avenue to be considered in the recent proposed involvement of chloride transporters in a number of developmentally related diseases, such as epilepsy, autism, and schizophrenia.

## Introduction

The ventral telencephalon is a major site of origin for both interneurons and macroglia cells (e.g., oligodendrocytes and astrocytes) of the cortex (Tekki-Kessaris et al., 2001; Kessaris et al., 2006). Improper timing of genesis as well as migration of these cell types has been implicated in a number of developmentally linked disorders, such as epilepsy, schizophrenia, and autism (Lewis, 2000; Powell et al., 2003; Rubenstein and Merzenich, 2003; Levitt et al., 2004).

In the developing ventral telencephalon, oligodendrocyte transcription factor 2 (Olig2)-labeled progenitor cells have the property to produce both oligodendrocytes and interneurons forming migratory streams toward the neocortex (Takebayashi et al., 2000; Miyoshi et al., 2007; Parras et al., 2007; Petryniak et al., 2007; Ono et al., 2008; Magalhães and Rivera, 2014). The mechanisms controlling the expression and expansion of Olig2 cell population are under active investigation.

The Na-K-Cl cotransporter isoform 1 (NKCC1) is a member of the cation-chloride cotransporter family, which plays a role in intracellular chloride regulation (Payne et al., 2003). The expression pattern of NKCC1 has been mainly studied in the developing telencephalon by *in situ* hybridization (Hübner et al., 2001; Li et al., 2002). These data showed that NKCC1 mRNA is expressed in the ventricular zone (VZ) and the subventricular zone (SVZ), which correspond to the proliferative zones (VZ/SVZ), of the lateral ganglionic eminence (LGE) at E12.5. Several studies have suggested the implication of NKCC1 in cell proliferation and cell cycle regulation *in vitro* (LoTurco et al., 1995; Haydar et al., 2000; Panet et al., 2000; Shiozaki et al., 2006). The high expression of NKCC1 in the developing ganglionic eminence (GE) points toward a possible role in cell proliferation and importance for the maintenance of progenitor cell population derived from this brain region. To this point, no investigation of NKCC1 in the cell cycle decision during embryonic brain development has been reported *in vivo*, and therefore its implication in the formation of interneuron and oligodendrocyte progenitor cells at embryonic stages has not been considered. For that purpose, we first studied the immuno-localization of NKCC1 in the developing telencephalon. We then analyzed its implication in cell cycle decision using NKCC1 knockout (KO) mice. Finally, we evaluated the consequences of the absence of NKCC1 expression on the formation of interneuron and oligodendrocyte progenitor cells. Here, we showed that NKCC1 is located in neural progenitor cells of the LGE at E12.5. We demonstrated that cell proliferation is reduced in the LGE in the absence of NKCC1 at the early stage of neurogenesis. The influence of NKCC1 in cell proliferation leads to an impairment in the formation of interneurons. Importantly, we also found that NKCC1 regulates the formation of Olig2-labeled oligodendrocyte progenitor cells. Thus, NKCC1 constitutes a modulator of neural progenitor cell proliferation through the cell cycle control at early stage of neurogenesis, and oligodendrogenesis.

## Materials and Methods

### Tissue Preparation

All animal experiments were approved by ELLA—The National Animal Experiment Board of Finland. Timed-pregnant mice were euthanized by CO_2_, and cervical dislocation. The day of vaginal plug was defined as embryonic day 0.5 (E0.5).

Embryos were used from C57BL/6N mice with disruption in *Slc12a2* gene encoding NKCC1 (Pace et al., 2000; Pfeffer et al., 2009). NKCC1 KO and wild-type (WT) were obtained by overnight mating of NKCC1 heterozygous mutant males and females. Genotyping was performed by polymerase chain reaction using standard protocols (Pfeffer et al., 2009). NKCC1 KO mouse embryos were compared with WT littermates used as controls.

Embryos were removed at E12.5, E13.5, E14.5, and E15.5, and fixed by immersion in 4% paraformaldehyde buffered with 0.1 M phosphate-buffered saline pH 7.4, at 4°C for 24–72 h.

For bromo-deoxyuridine (BrdU)-incorporation analysis, time-pregnant mice were intraperitoneally injected with the cell proliferation labeling reagent containing BrdU (10 mM, 3 mg/ml) and fluoro-deoxyuridine (1 mM, 0.3 mg/ml), (2 ml/100 g, Amersham Pharmacia). Cell proliferation labeling reagent was administrated to mutant females (*n* = 5) for 30 min at E12.5 and at E14.5, for 2 h at E12.5, and for 24 h at E12.5 and at E14.5.

### Immunohistochemistry

Immunohistochemistry for paraffin embedded-sections using heat-induced epitope retrieval methods were performed as previously described (Magalhães and Rivera, 2014).

The primary antibodies used in this study were as follows: rat anti-BrdU (1:400, OBT0030G, AbD Serotec), rabbit anti-cleaved caspase-3 (1:50, 9661, Cell Signaling), guinea-pig anti-Dlx2 (distal-less homeobox 2; 1:3000, gift from Dr. Kazuaki Yoshikawa, Osaka University, Osaka, Japan), rabbit anti-Ki67 (1:400, RM-9106-S0, Thermo Fisher Scientific), mouse anti-Nestin (1:400, MAB353, Merck Millipore), rabbit anti-NKCC1 α-wNT (1:200, gift from Dr. Robert James Turner), rabbit anti-Olig2 (1:500, AB9610, Merck Millipore), rabbit anti-phospho-Histone H3 (PH3; 1:1000, 06-570, Merck Millipore), goat anti-Sp8 (1:500, sc-104661, Santa Cruz Biotechnology), mouse anti-TuJ1 (1:5000, MMS-435P, Covance Laboratories).

The secondary antibodies used in this study were as follows: Goat anti-rat Alexa Fluor 568 (1:400, A-11077), Goat anti-guinea-pig Alexa Fluor 568 (1:400, A-11075), Donkey anti-mouse Alexa Fluor 488 (1:400, A-21202), Goat anti-rabbit Alexa Fluor 568 (1:400, A-11011), Donkey anti-goat Alexa Fluor 568 (1:400, A-11057, Thermo Fisher Scientific); Goat anti-rabbit Dylight 488 (1:200, FDR488), Goat anti-rabbit Dylight 549 (1:200, FDR549, Biocare Medical).

### Image Acquisition

Samples were analyzed on Zeiss AX10 fluorescence microscope, with the Zeiss Plan-APOCHROMAT 10× and 40× objectives. Higher magnification images were obtained on Zeiss LSM5 confocal microscope, with the Zeiss Plan-APOCHROMAT 63× objective. Images were processed using Adobe Photoshop CS6 software to adjust brightness and contrast.

### Data Analysis

The number of PH3-labeled mitotic cells was manually measured by the cell counter feature in Adobe Photoshop CS6. VZ cells lining the ventricular surface were counted within 1 mm.

Apical and basal cleaved caspase-3-labeled cells were manually counted in the VZ lining the ventricular surface and in a region of interest (400 × 400 μm) drawn throughout the VZ/SVZ, respectively.

The integrated density of labeling from BrdU, Ki67, and Dlx2 was measured in ImageJ 1.47v software (NIH, Bethesda, MD, USA).

Analyses of cell cycle parameters were performed according to Chenn and Walsh (2002). For cell cycle length analysis, E12.5 NKCC1 heterozygous mice were treated with BrdU for 30 min, and followed by co-labeling of BrdU together with Ki67 in brain sections. Percentage of S-phase cells labeled with BrdU, defined as the cell cycle length index, were determined by the integrated density of BrdU-labeling divided by the integrated density of Ki67-labeling (BrdU-positive/Ki67-positive labeling) in the LGE. Decreased cell cycle length index suggests lengthened cell cycle. For cell cycle reentry analysis, E12.5 NKCC1 heterozygous mice were treated with BrdU for 24 h, and followed by co-labeling of BrdU together with Ki67 in brain sections. Cells that left the cell cycle were identified as BrdU-positive and Ki67-negative, and cells that reentered the cell cycle were identified as BrdU-positive and Ki67-positive. Percentage of cells that reentered the cell cycle, defined as the cell cycle reentry index, was determined by the integrated density of Ki67-labeling divided by the integrated density of BrdU-labeling (Ki67-positive/BrdU-positive labeling) in the LGE.

Data are presented as mean fold change ± standard error mean (SEM) relative to WT embryos.

The length of labeling from TuJ1, Olig2, and Sp8 was measured in ImageJ 1.47v. The length of the region containing either TuJ1- or Olig2-labeling was measured along a line perpendicular to the middle of the ventricular surface in the LGE of NKCC1 KO and WT mice. The length of the region containing Sp8-labeling was measured along a line extending from the SVZ of dorsal LGE of NKCC1 KO and WT mice.

Data are presented as mean ± SEM relative to WT embryos.

Statistical analyses were performed using the two-tailed Student’s *t*-test with a value of *p* < 0.05 considered as significant, in SigmaPlot software version 11.0 (Systat Software, Erkrath, Germany).

## Results

### NKCC1-Expressing Cells are VZ/SVZ Cells in the Ventral Telencephalon

**Figure 1:**
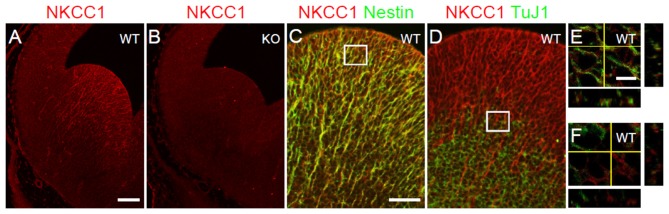
**Na-K-Cl cotransporter (NKCC1) is expressed in proliferative zone cells. (A,B)** Immunohistochemical staining for NKCC1 in the lateral ganglionic eminence (LGE) of wild-type (WT) **(A)** and NKCC1 knockout (KO) (*n* = 6) **(B)** mice at E12.5. **(C–F)** Co-labeling of NKCC1 together with nestin (*n* = 3) **(C)**, and with TuJ1 (*n* = 3) **(D)** in E12.5 WT LGE. **(C)** The merge image shows the co-localization of NKCC1 and nestin. **(D)** The merge image shows the opposing expression patterns of NKCC1 and TuJ1. High-magnification confocal images of the boxed area in **(C,D)** are shown in **(E,F),** respectively. Scale bar 200 μm in **(A)**, 100 μm in **(C)**, 20 μm in **(E)**.

Previous studies have reported that NKCC1 mRNA is detected in the VZ/SVZ of LGE at E12.5 (Hübner et al., 2001; Li et al., 2002). Here, we examined the expression pattern of NKCC1 protein by performing immunohistochemical staining using NKCC1 antibody on coronal sections of E12.5 brains from WT and NKCC1 KO mice. We observed that NKCC1 was detected in the VZ/SVZ of E12.5 LGE (Figure 1A). The specificity of NKCC1 antibody was confirmed by the absence of staining in the LGE of NKCC1 KO mice (Figure 1B). Furthermore, NKCC1 expression in the LGE coincided with the expression of nestin, an intermediate filament protein known as a neural progenitor cell marker, suggesting that NKCC1 is expressed by the progenitor cell pool (Figures 1C,E). Consistently, NKCC1 and TuJ1 (neuron-specific class III β-tubulin), a marker of immature neurons, displayed opposite expression patterns in the LGE region: only a few NKCC1 and TuJ1 co-labeled cells were located in the intermediate zone (Figures 1D,F). These data indicate that NKCC1-expressing cells are neural progenitor cells in the VZ/SVZ of the mouse LGE.

### Mice Lacking NKCC1 Exhibits Abnormal Gross Morphology of the LGE

**Figure 2:**
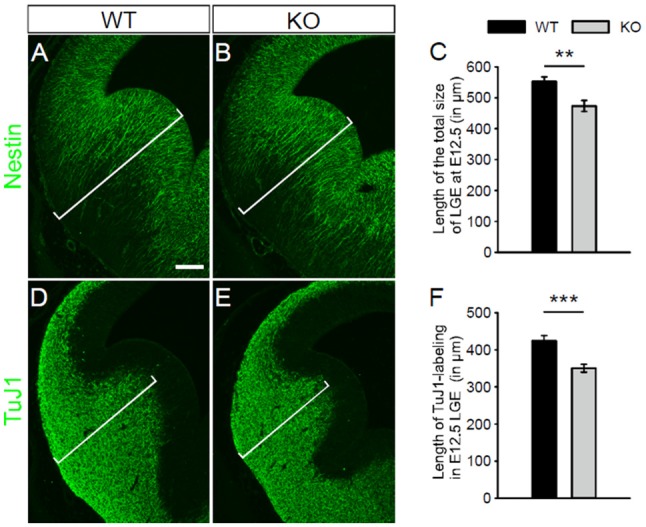
**NKCC1-deficient mice exhibit abnormal morphology of the LGE. (A,B)** Immunohistochemical staining for nestin in the LGE of WT **(A)** and NKCC1 KO **(B)** mice at E12.5. **(C)** Quantification of the length of the total size of the LGE in WT and NKCC1 KO mice at E12.5. **p < 0.005 (*n* = 4). **(D,E)** Immunohistochemical staining for TuJ1 in the LGE of WT **(D)** and NKCC1 KO **(E)** mice at E12.5. **(F)** Quantification of the length of the region containing TuJ1-labeling in the LGE of WT and NKCC1 KO mice at E12.5. ***p < 0.001 (*n* = 8). Scale bar 200 μm in **(A)**.

Pfeffer et al. (2009) have reported that no alteration of the gross structure of the cerebral cortex is observed in NKCC1 KO mice at postnatal stages. At E12.5, we examined the gross morphology of the LGE in NKCC1 KO and WT mice, assessed by nestin- (Figures 2A,B) and TuJ1- (Figures 2D,E) labeling. We observed that mice lacking NKCC1 displayed morphology abnormality in the LGE: the LGE was smaller in the NKCC1 KO than in the WT mice (Figures 2A,B,D,E). The reduced morphology of the LGE was evaluated quantitatively by measuring the length of the total size of the LGE in nestin-labeled sections, and the mantle region containing TuJ1-labeled cells. This analysis showed a significant reduction in the LGE (Figure 2C), and in the mantle region of NKCC1 KO relative to WT mice (Figure 2F). These data indicate that mice lacking NKCC1 exhibit smaller LGE than of the WT mice at E12.5.

### NKCC1-Expressing Cells are Proliferating Cells in the Ventral Telencephalon

**Figure 3:**
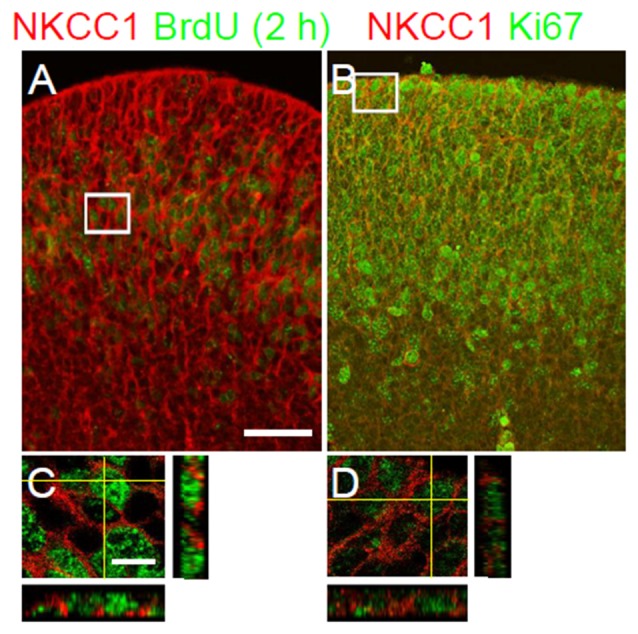
**NKCC1-expressing cells are proliferating cells in the proliferative zones. (A,B)** Co-labeling of NKCC1 together with bromo-deoxyuridine (BrdU; *n* = 2) **(A)**, and with Ki67 (*n* = 2) **(B)** in the ganglionic eminence at E12.5. The merge images show the co-localization of NKCC1 and BrdU **(A)**, and Ki67 **(B)**. High-magnification confocal images of the boxed area in **(A,B)** are shown in **(C,D)**, respectively. Scale bar 100 μm in **(A)**, 20 μm in **(C)**.

The distribution of NKCC1 expression and the abnormal gross morphology of the LGE in the NKCC1-deficient mice prompted us to consider a potential role of NKCC1 in the proliferation of VZ/SVZ cells. To assess this hypothesis, we treated E12.5 NKCC1 heterozygous mice with BrdU for 2 h. Then, we performed co-labeling of NKCC1 together with BrdU, which labels cells at S-phase, on E12.5 brain sections. Pulse labeling of BrdU showed that NKCC1 expression corresponded to both BrdU-positive and BrdU-negative cells in the VZ/SVZ of LGE at E12.5 (Figures 3A,C). In addition, we performed co-labeling of NKCC1 together with Ki67, which labels cells at all active stages of the cell cycle, on E12.5 brain sections. NKCC1 expression corresponded to Ki67-positive cells in the VZ/SVZ of GE at E12.5 (Figures 3B,D). These data indicate that NKCC1-expressing cells are proliferating cells in the VZ/SVZ of E12.5 mouse.

### NKCC1 is Involved in Cell Proliferation and Cell Cycle Reentry

**Figure 4:**
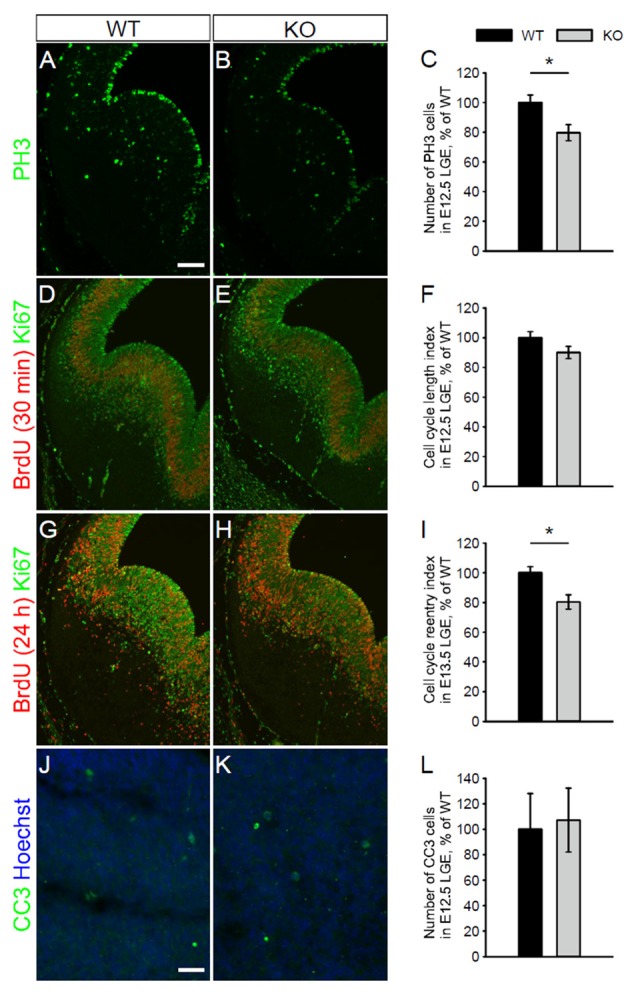
**NKCC1 regulates cell proliferation *in vivo* by controlling cell cycle decision. (A,B)** Immunohistochemical staining for phospho-Histone H3 (PH3) in the LGE of WT **(A)** and NKCC1 KO **(B)** mice at E12.5. **(C)** Quantification of PH3-labeled mitotic cells in the ventricular zone (VZ) lining the ventricular surface of WT and NKCC1 KO mice at E12.5. *p < 0.05 (*n* = 6). **(D,E)** Co-labeling of BrdU together with Ki67 in the LGE of WT **(D)** and NKCC1 KO **(E)** mice at E12.5. **(F)** Quantification of cell cycle length index in the LGE of WT and NKCC1 KO mice at E12.5. *p* > 0.05 (*n* = 4). **(G,H)** Co-labeling of BrdU together with Ki67 in the LGE of WT **(G)** and NKCC1 KO **(H)** mice at E13.5. **(I)** Quantification of cell cycle reentry index in the LGE of WT and NKCC1 KO mice at E13.5. *p < 0.05 (*n* = 4). **(J,K)** Co-labeling of cleaved caspase-3 (CC3) together with Hoechst in the LGE of WT **(J)** and NKCC1 KO **(K)** mice at E12.5. **(L)** Quantification of CC3-labeled cells in the LGE of WT and NKCC1 KO mice at E12.5. *p* > 0.05 (*n* = 4). Error bars in **(C,F,I,L)** indicate standard error mean (SEM). Scale bar 200 μm in **(A)**, 50 μm in **(J)**.

We next assessed whether the absence of NKCC1 leads to changes in cell proliferation by monitoring the pattern of mitotic cells. For that purpose, we first examined the density of PH3-labeled cells close to the ventricular surface of LGE from WT and NKCC1 KO mice at E12.5. We found that the density of progenitor cells in mitotic phase was reduced in the VZ of LGE from NKCC1 KO relative to WT brains (Figures 4A–C). As this could be derived from increase cell apoptosis, we estimated whether there were changes in the cleaved caspase-3 (CC3)-labeling. No increase in CC3-labeled cells was detected in the VZ/SVZ of LGE from NKCC1 KO relative to WT brains (Figures 4J–L). We then addressed whether the decrease in proliferating cells is caused by lengthening of the cell cycle, or whether it is the result of impairment in cell cycle reentry. For that purpose, we examined BrdU incorporation and Ki67 expression in the LGE of WT and NKCC1 KO brains at E12.5. The rate of proliferation was analyzed in the LGE by performing a BrdU pulse-chase experiment (a 30 min BrdU pulse) combined with Ki67 staining, and measuring the integrated density of BrdU- and Ki67-labeling. The ratio between the integrated densities of BrdU- and Ki67-labeling gives an estimation of the cell cycle length. No significant difference in cell cycle length index appeared in NKCC1 KO relative to WT brains (Figures 4D–F). We then analyzed the cell cycle reentry in LGE by performing a BrdU pulse-chase experiment (a 24 h BrdU pulse) combined with Ki67 staining, and measuring the integrated density of Ki67- and BrdU-labeling. The ratio between the integrated densities of Ki67- and BrdU-labeling gives an estimation of the cell cycle reentry. We observed a significant decrease in cell cycle reentry index in NKCC1 KO relative to WT brains (Figures 4G–I). Together, these data demonstrate that the absence of NKCC1 significantly affects the proliferation of VZ cells and the cell cycle regulation in E12.5 LGE, suggesting that NKCC1 is involved in cell proliferation in the ventral telencephalon.

### NKCC1 is Involved in the Development of Interneurons and Oligodendrocytes

**Figure 5:**
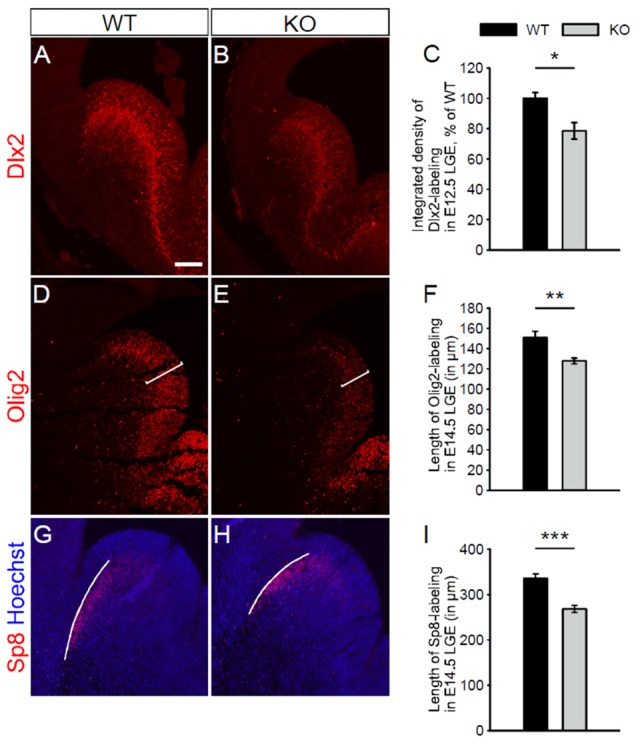
**NKCC1 is involved in the development of interneurons and oligodendrocytes. (A,B)** Immunohistochemical staining for distal-less homeobox 2 (Dlx2) in the LGE of WT (in **A**) and NKCC1 KO (in **B**) mice at E12.5. **(C)** Quantification of Dlx2 integrated density in the LGE of WT and NKCC1 KO mice at E12.5. *p < 0.05 (*n* = 4). **(D,E)** Immunohistochemical staining for Olig2 in the LGE from WT **(D)** and NKCC1 KO **(E)** mice at E14.5. **(F)** Quantification of the length of the region containing Olig2-labeling in the LGE of WT and NKCC1 KO mice at E14.5. **p < 0.005 (*n* = 4). **(G,H)** Co-labeling of Sp8 together with Hoechst in the LGE from WT **(G)** and NKCC1 KO **(H)** mice at E14.5. **(I)** Quantification of the length of the region containing Sp8-labeling in the LGE of WT and NKCC1 KO mice at E14.5. ***p < 0.001 (*n* = 4). Error bars in **(C,F,I)** indicate SEM. Scale bar 200 μm in **(A)**.

Petryniak et al. (2007) have shown that Dlx2 and Olig2, as markers of interneuron and oligodendrocyte progenitor cells respectively, are transiently co-expressed in progenitor cells within the developing ventral telencephalon. Waclaw et al. (2006) have also shown that Sp8 (a member of the Sp1 zinc finger transcription factor gene family), as a marker of immature interneurons, is expressed in interneurons migrating from the dorsal LGE. To assess whether the absence of NKCC1 affects the formation of interneuron and oligodendrocyte progenitor cells, we examined the expression of these different markers Dlx2, Olig2, and Sp8 in the LGE at different embryonic stages. At E12.5, we observed a reduced expression of Dlx2 in the LGE of NKCC1 KO relative to WT brains (Figures 5A–C). Additionally, we performed co-labeling of PH3 together with Dlx2 at E12.5. The density of PH3 and Dlx2 co-labeled cells is decreased in the ventricular surface of NKCC1 KO relative to WT brains (WT: 100 ± 11%; KO: 69 ± 10%; *p* < 0.05; *n* = 6), suggesting that the decrease in Dlx2-labeled progenitor cells is due to a decrease in cell proliferation in E12.5 LGE.

As oligodendrocyte progenitor cells have a delayed developmental appearance, we performed immunohistochemical staining at a later embryonic stage, using markers for immature interneurons, and immature oligodendrocytes. Dlx2 expression was not affected in E14.5 NKCC1 KO brains (WT: 100 ± 3%; KO: 153 ± 29%; *p* > 0.05; *n* = 4). Interestingly, the NKCC1 KO LGE displayed less expression of Olig2 than WT brains at E14.5 (Figures 5D,E). The reduced thickness of Olig2-labeling was evaluated quantitatively by measuring the length of the region containing Olig2-labeled cells. This analysis disclosed a reduced expression of Olig2 in the LGE of NKCC1 KO relative to WT brains (Figure 5F). Interestingly, no difference appeared in the density of PH3 and Olig2 co-labeled cells in the ventricular surface of NKCC1 KO relative to WT brains (WT: 100 ± 11%; KO: 102 ± 14%; *p* > 0.05; *n* = 4), suggesting that the decrease in Olig2-labeled progenitor cells is not due to a decrease in proliferation at this developmental stage in the LGE. In order to study the impact of decrease proliferation at E12.5 in NKCC1 KO on LGE-derived interneuron population, we monitored migrating Sp8-labeled immature interneurons by measuring the length of the region containing Sp8-labeling at E14.5. Here, we observed a reduction in the wave of migrating cells in the dorsal LGE (Figures 5G–I). Together, these data indicate that NKCC1 is involved in the formation of both interneuron and oligodendrocyte progenitor cells within the LGE.

## Discussion

Previous studies in the postnatal brain have shown an important implication of NKCC1 in the maturation of synaptic transmission and network activity (Pfeffer et al., 2009). However, little is known about its implication in the development of interneurons and oligodendrocytes. In this article, we have identified *in vivo* the implication of NKCC1 in the proliferation of neural progenitor cells from the LGE at an early stage of neurogenesis (Magalhães, 2016). The data indicates that the mechanism for this effect involved regulation of cell cycle reentry. In addition, these results also indicate that NKCC1 is implicated in the development of interneurons and oligodendrocytes.

Several studies have defined in the developing embryonic brain that NKCC1 mRNA is expressed in the VZ/SVZ of LGE at E12.5 (Hübner et al., 2001; Li et al., 2002). Li et al. (2002) used NKCC1 T4 antibody to detect NKCC1 protein in the VZ/SVZ of rat ventral telencephalon at E14.5. However, the specificity of NKCC1 antibody was not validated in NKCC1 KO brains. Here, we used NKCC1 α-wNT antibody to detect NKCC1. The specificity of this antibody has been previously confirmed in NKCC1 KO tissues at postnatal stages (Mao et al., 2012). In the present study, the specificity of this antibody in immunohistochemistry was further validated in NKCC1 KO embryonic mouse brain sections. Thus, this is the first study of NKCC1 immunohistochemical detection in the embryonic mouse brain using a validated antibody. Using this antibody, we showed that NKCC1 protein is expressed in the VZ/SVZ of mouse LGE at E12.5. Consistently, NKCC1 co-localized with nestin, and its expression was low in TuJ1-labeled region. In accordance with the expression of NKCC1 in the VZ/SVZ, we found that NKCC1 co-localized with cell cycle markers. Thus, NKCC1-expressing cells are neural progenitor cells in the VZ/SVZ of the E12.5 mouse LGE.

It has been reported that no morphological alterations were detected in the postnatal cerebral cortex of NKCC1 KO mice (Pfeffer et al., 2009). However, no anatomical analysis of the LGE has been performed previously. In this present study, we found a significant reduction in the total size of the LGE and its mantle region in E12.5 NKCC1 KO mice. In line with this data, other studies have reported an implication of NKCC1 in the development of cortical neurons, cortical and cerebellar oligodendrocytes (Young et al., 2012; Fu et al., 2015; Zonouzi et al., 2015). Together, these results indicate that the *in vivo* impact of NKCC1 expression in brain development may depend on the brain region. Furthermore, the abnormal morphology of the LGE in the NKCC1 KO as well as the expression of NKCC1 in the neural progenitor cells suggests that NKCC1 may regulate the proliferation of these cells in the developing telencephalon. Previous studies have indicated the implication of NKCC1 in cell cycle regulation by its overexpression and inhibition with the specific loop diuretics, bumetanide and furosemide, using immortalized cell lines and primary cell cultures (Panet et al., 2000; Shiozaki et al., 2006).

NKCC1 is known to increase intracellular chloride concentration, thereby promoting depolarization of immature neurons via γ-aminobutyric acid (GABA)_A_-receptor activation (Blaesse et al., 2009). GABA can act as a regulator of cell proliferation (LoTurco et al., 1995; Fiszman et al., 1999; Haydar et al., 2000). LoTurco et al. (1995) have shown that GABA-dependent depolarizing effect decreases DNA synthesis in E16–E17 rat VZ/SVZ cortical cells. Here, we found *in vivo* that mice lacking NKCC1 exhibited decreased mitotic activity of VZ cells at E12.5, indicating that cell proliferation in LGE involves NKCC1 expression. This result contrasts with LoTurco findings, in which GABA negatively regulates cell proliferation in the rat neocortex at E16–E17. However, GABA increases immature granule cell proliferation in the rat cerebella at postnatal day 6–7 (Fiszman et al., 1999). The effect of GABA on cell proliferation appears to be cell type- and time-dependent. We further found that the decrease in cell proliferation was not due to the lengthening of cell cycle, but to the impairment of cell cycle reentry in the LGE at E12.5, suggesting that NKCC1 is involved in the modulation of cell cycle decision in the developing ventral telencephalon. Thus, NKCC1 may function in neural progenitor cells to influence their decision to reenter the cell cycle. This, in turn, regulates the differentiated cell pool at E12.5 in this region. However, the mechanism for NKCC1-mediated cell cycle regulation remains to be elucidated. It has been reported that NKCC1 increases the volume of proliferating cells before they enter the S-phase (Habela et al., 2009). NKCC1 also maintains high intracellular chloride concentration in immature cortical neurons, which leads to GABA-induced membrane depolarization (Yamada et al., 2004; Achilles et al., 2007). Studies have shown that GABA (LoTurco et al., 1995; Fiszman et al., 1999; Haydar et al., 2000) and NKCC1 (Panet et al., 2000) modulate cell proliferation via MAPK pathway *in vitro* (Fiszman et al., 1999; Panet et al., 2002; Fu et al., 2015). Monitoring the phosphorylation of MAPK as well as cell cycle-regulated proteins and related transcription factors could provide a molecular mechanism for NKCC1-mediated cell cycle progression in the LGE. Thus, NKCC1 may have a permissive role in regulating cell volume or target proteins to control the proliferation of neural progenitor cells in the LGE *in vivo*.

In the developing ventral telencephalon, as interneurons and oligodendrocytes share a common progenitor population (He et al., 2001; Yung et al., 2002; Petryniak et al., 2007), the expression of NKCC1 in the LGE points towards a possible role in the formation of interneurons and oligodendrocytes. The formation of these cells depends on Dlx2 and Olig2 that are co-expressed in neural progenitor cells in the ventral telencephalon (Petryniak et al., 2007). Dlx2 is expressed in the VZ/SVZ of LGE and MGE at E12.5 (Eisenstat et al., 1999; Petryniak et al., 2007). Olig2 is detected in the VZ/SVZ of LGE and mostly in MGE at E12.5, and then in both the LGE and MGE at E14.5 (Takebayashi et al., 2000; Petryniak et al., 2007; Ono et al., 2008; Magalhães and Rivera, 2014). Consistently, Dlx2 was expressed in the VZ/SVZ of LGE from WT mice at E12.5 and E14.5. We found that Dlx2 expression was reduced in E12.5 LGE of mice lacking NKCC1 compared with WT brains. We further showed that the mitotic activity within Dlx2 population was lower in the LGE of NKCC1 KO compared with WT brains, indicating that the regulation of Dlx2 expression depends on cell proliferation at E12.5. Once VZ cells exit the cell cycle, they adopt a neuronal or glial fate, and start migrating within the developing telencephalon. Interestingly, Sp8 expression was reduced in E14.5 LGE of mice lacking NKCC1 compared with WT brains, indicating that NKCC1 is involved in the development of interneurons that migrate from the LGE. These observations demonstrate that NKCC1 is involved in the formation of interneuron progenitor cells at the early stage of neurogenesis leading to less migrating interneurons from the dorsal LGE at a later stage. Consistently, Young et al. (2012) have shown that knocking down NKCC1 reduced progenitor cell proliferation in the SVZ of newborn mice, thereby reducing neuronal production. No change in cell apoptosis was detected in the SVZ-olfactory bulb axis (Young et al., 2012). At E14.5, oligodendrocyte precursor cells are born in the LGE (Kessaris et al., 2006). At E14.5, no difference appeared in Dlx2 expression in the LGE of NKCC1 KO compared with WT brains, indicating that Dlx2 expression is only affected at early stage of neurogenesis. At the onset of oligodendrogenesis, Olig2 expression was reduced in the LGE of mice lacking NKCC1 compared with WT brains. Furthermore, no difference appeared in the mitotic activity within Olig2 population in the LGE of NKCC1 KO compared with WT brains, indicating that the regulation of Olig2 expression requires NKCC1 at E14.5. In newborn rats, inhibiting NKCC1 decreases cell cycle progression arrest and reduction in the proliferation of oligodendrocyte precursor cells under oxygen-glucose deprivation conditions, by mediating the expression of cell cycle regulators (Fu et al., 2015). Furthermore, knocking down NKCC1, that reduces neural progenitor cell proliferation and neuronal production, delays dendritic morphogenesis (Young et al., 2012). Therefore, NKCC1 may regulate the development of neurons and oligodendrocytes depending on the timing and region of expression.

Together, these data indicate the importance of NKCC1 expression in the cell division of the proliferative progenitor pool of the LGE. This is the first report to show the involvement of NKCC1 in the development of the neurogenic and oligodendrogenic pool of this region. Recent reports have linked NKCC1 to neurodevelopmental disorders, such as epilepsy and schizophrenia (Morita et al., 2014; Marguet et al., 2015). Abnormal development of interneurons and oligodendrocytes have been proposed to be involved in these disorders (Chen et al., 2015; Wang et al., 2015; Wu and Sun, 2015). Thus, the implication of NKCC1 in the LGE presented in this study may be important in the etiology of neurodevelopmental disorders.

## Conclusion

The ventral telencephalon is a main source for both cortical interneurons and oligodendrocytes. Abnormal number as well as timing of genesis may result in detrimental developmental phenotypes that may underlay a number of disorders such as epilepsy, autism, and schizophrenia. In the present study, we indicate that NKCC1 is expressed in the VZ/SVZ of the mouse LGE at the early stage of neurogenesis. We report that NKCC1 is involved in cell proliferation by influencing neural progenitor cells to reenter the cell cycle. Importantly, we show that NKCC1 is involved in the development of immature interneurons and oligodendrocytes.

## Author Contributions

ACM designed and performed the experiments, analyzed the data, and wrote the manuscript. CR designed the experiments and wrote the manuscript.

## Conflict of Interest Statement

The authors declare that the research was conducted in the absence of any commercial or financial relationships that could be construed as a potential conflict of interest.
